# Does direction of results of abstracts submitted to scientific conferences on drug addiction predict full publication?

**DOI:** 10.1186/1471-2288-9-23

**Published:** 2009-04-08

**Authors:** Simona Vecchi, Valeria Belleudi, Laura Amato, Marina Davoli, Carlo A Perucci

**Affiliations:** 1 Department of Epidemiology, Italian National Health Service, Local Health Unit Rome E, Via di S. Costanza, 53, 00198 Rome, Italy

## Abstract

**Background:**

Data from scientific literature show that about 63% of abstracts presented at biomedical conferences will be published in full. Some studies have indicated that full publication is associated with the direction of results (publication bias). No study has looked into the occurrence of publication bias in the field of addiction.

**Objectives:**

To investigate whether the significance or direction of results of abstracts presented at the major international scientific conference on addiction is associated with full publication

**Methods:**

The conference proceedings of the US Annual Meeting of the College on Problems of Drug Dependence (CPDD), were handsearched for abstracts of randomized controlled trials and controlled clinical trials that evaluated interventions for prevention, rehabilitation and treatment of drug addiction in humans (years searched 1993–2002). Data regarding the study designs and outcomes reported were extracted. Subsequent publication in peer reviewed journals was searched in MEDLINE and EMBASE databases, as of March 2006.

**Results:**

Out of 5919 abstracts presented, 581 met the inclusion criteria; 359 (62%) conference abstracts had been published in a broad variety of peer reviewed journals (average time of publication 2.6 years, SD +/- 1.78). The proportion of published studies was almost the same for randomized controlled trials (62.4%) and controlled clinical trials (59.5%) while studies that reported positive results were significantly more likely to be published (74.5%) than those that did not report statistical results (60.9%.), negative or null results (47.1%) and no results (38.6%), Abstracts reporting positive results had a significantly higher probability of being published in full, while abstracts reporting null or negative results were half as likely to be published compared with positive ones (HR = 0.48; 95%CI 0.30–0.74)

**Conclusion:**

Clinical trials were the minority of abstracts presented at the CPDD; we found evidence of possible publication bias in the field of addiction, with negative or null results having half the likelihood of being published than positive ones.

## Background

Dissemination of new scientific research is a critical issue for researchers. Improving dissemination helps to accelerate research, to enrich education, and to enhance return on taxpayer investment in research. Usually international meetings are the first step in this process of dissemination, when preliminary results are presented, eventually followed by full publication of the study in a peer-reviewed journal. How and when the research is published can be as significant as the research results themselves, since the influence of a research article may only be as potent as its ability to attract an audience of readers.

Data from a systematic review [1] of reports that examine the rate of full publication of results presented as abstracts show that 63% of abstracts presented at biomedical meetings will be published in full and that positive study outcomes are more likely to be subsequently published. This results in a particular form of bias known as publication bias; this bias has a particular impact on the quality of systematic reviews, which involve the comprehensive and unbiased identification of relevant studies. When this bias exists, systematic reviews will tend to over-estimate treatment effects.

Another systematic review on the impact of including grey literature in meta-analyses of healthcare interventions [2] showed that published trials tend to be larger and show an overall greater treatment effect than unpublished trials. This is important particularly in fields where only a few small trials of a health care intervention have been carried out.

We set about to investigate the occurrence of publication bias in the field of addiction, focusing on the direction of the results and their association with full publication of abstracts presented at the Annual Meeting of College on Problems of Drug Dependence (CPDD), one of the most important international scientific conferences on addiction.

## Methods

### Identification of the studies

We considered the abstracts presented at the Annual Meeting of College on Problems of Drug Dependence (CPDD), US, between 1993 and 2002 (N = 5919) which were published as supplements in the Drug and Alcohol Dependence journal. Abstracts from other international conferences were not available. We handsearched and screened the titles and the abstracts for RCTs and CCTs, evaluating interventions for prevention, rehabilitation and treatment of drug addiction in humans. Trials were classified as RCTs when the randomization was explicitly defined and as CCTs when it was not.

### Exclusion criteria

We excluded abstracts reporting analyses on healthy people, pharmacokinetic and toxicity studies, and preliminary findings reporting the characteristics of the participants without mention of the allocation groups.

### Data extraction

Data was selected by one investigator (SV). For each selected abstract we collected information on the year of the meeting, study design, country, substance of abuse and results. We classified the results of each trial according to the primary outcome as:

▪ Positive results: statistically significant results (p < 0.05) in the experimental arm.

▪ Negative or Null results: statistically significant results (p < 0.05) in the control arm, or not statistically significant (p = >0.05) results.

▪ Not reported statistical results: abstracts that did not report statistical significance.

▪ No results: abstracts that do not provide any results.

### Literature searches

Full-length articles published in peer reviewed journals were searched in MEDLINE and EMBASE electronic databases from 1992 to March 2006, with no language restrictions. The first search criterion was the combination of the first author's name and/or keywords in the title or abstract. When this search strategy did not identify any publications, we added the subsequent authors' names to the search. We considered the abstract as published if a) at least one author of the abstract was an author of the full publication and b) the primary outcome from the abstract was an outcome in the full manuscript. When a publication was confirmed, we recorded the journal, month and year of publication. For journals published in the spring or fall we assigned the months March or October, respectively.

If the abstract was published more than once, we used the earliest publication in the analysis. Abstracts published in full before the presentation at the Conference were excluded.

### Statistical analysis

To evaluate the association between the study findings and the time interval between submission and publication, we performed a Kaplan Meier analysis and estimated hazard ratios of publication. We also examined the association between characteristics of the study (i.e. substance of abuse) and full publication. Person-time at risk for full publication was computed as time since presentation of the abstract to the Conference till time of full publication or end of follow-up (March 2006).

## Results

Out of a total of 5919 abstracts submitted, 581 met the inclusion criteria; 359 (62%) were subsequently published in peer reviewed journals, of which 284 were reports of RCTs. Table 1 shows the number of abstracts identified and the percentage of them published in full, by year.

**Table 1:** Abstracts of controlled trials published in full, by year.

Conference Year	# abstracts identified	# abstracts published (%)
1993	59	37 (62.7)
1994	50	34 (68.0)
1995	51	13 (25.5)
1996	27	17 (63.0)
1997	41	26 (63.4)
1998	58	44 (75.9)
1999	60	40 (66.7)
2000	80	48 (60.0)
2001	83	56 (67.5)
2002	72	44 (61.1)
**Total**	**581**	**359 (62.0)**

Of the abstracts published in full, the most common substances of abuse considered were opioids (127 trials, 35.4%), cocaine (106 trials, 29.5%), not-specified (42 trials, 11.7%), poly-abuse (36 trials, 10%) and cannabis (17 trials, 4.7%). Other substances of abuse (amphetamines, alcohol, benzodiazepines, caffeine, etc.) were considered in less than 10% of the studies.

The abstracts were published in 57 journals: 21% in Drug and Alcohol Dependence, 7.6% in the Journal of Consulting and Clinical Psychology and in Psychopharmacology, 6.3% in the Journal of Substance Abuse Treatment and Experimental & Clinical Psychopharmacology, and the remaining 51.2% in a variety of other journals (with < = 3% in a single journal).

Table 2 shows the various study designs and results with their corresponding publication rates. The proportion of studies published was split roughly equally for RCTs (62.4%) and CCTs (59.5%) while studies that reported positive results were significantly more likely to be published (74.5%) than those that did not report statistical results (60.9%), negative or null results (47.1%) and no results (38.6%). Abstracts reporting positive results had a significantly higher probability of being published in full, while abstracts reporting null or negative results were half as likely to be published compared with positive ones (Table 3).

**Table 2:** Distribution of the presented abstracts by study design and direction of results

	Published N (%)	Not Published N (%)	Total N	OR	CI 95%	
						

**Study design**						
RCT	284 (62.4)	171 (37.5)	455	1.00		
CCT	75 (59.5)	51 (40.5)	126	0.9	0.59	1.33
**Results**						
Positive results	120 (74.5)	41 (25.5)	161	1.00		
No reported statistical results	198 (61)	127 (39)	325	0.5	0.4	0.8
Negative/null results	24 (47.1)	27 (52.9)	51	0.3	0.2	0.6
No results	17 (38.6)	27 (61.4)	44	0.2	0.1	0.4
**Total**	**359**	**222**	**581**			

**Table 3:** Rate of full publication by study results

	Trials published	Median time of publication (years)	follow-up 100 years	Rate	Hazard Ratio	CI 95%	
Positive	120	2.9	6.5	18.5	1.00		
Not reported	198	4.2	16.2	12.2	0.70	0.56	0.9
Null or negative	24	5.2	3.0	8.0	0.48	0.30	0.74
No results	17	5.2	2.2	7.7	0.38	0.23	0.64

The median time lapse until publication was 3.8 years, The time duration from presentation at the conference to publication was shorter for trials with positive results than it was for trials with negative or null results (a median of 2.9 years vs 5.2 years, respectively; p < 0.001) (see Table 3).

**Figure 1:**
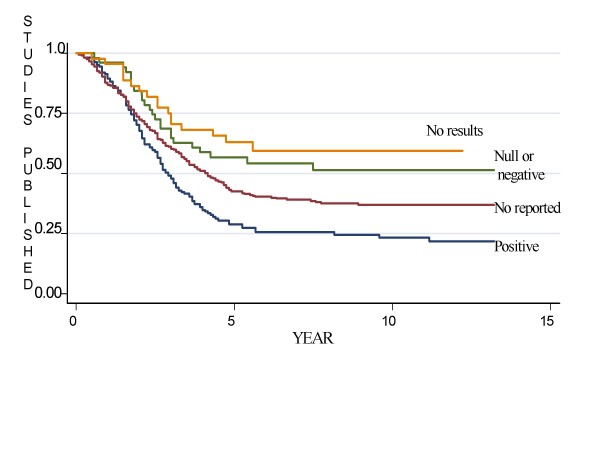
**The time-dependent probability (Kaplan Meier survival estimates) of publication of studies based on their results**.

The time-dependent probability of publication of studies based on their results is presented in Figure 1. We found evidence that the likelihood of publication differed by study results (Log-rank test p < 0.001).

## Discussion and Conclusion

The results of this study demonstrate that RCTs and CCTs evaluating the effectiveness of interventions for drug addiction represent a minor proportion of all abstracts presented at the CPDD Conference. Overall, 62% of abstracts presented at the CPDD meeting based on randomised or clinically controlled trials were subsequently published in full in peer reviewed journals. This percentage is consistent with other studies on publication rates of abstracts presented at international conferences in different areas of medicine [1,3-8]. No previous studies were available in the area of addiction.

Our results also indicate that studies with positive findings are more likely to be published. These results are similar to those from the study by Scherer et al. [1], who conducted a meta-analysis combining data from eight reports that examined full publication of abstracts describing randomized or controlled trials and found an association between 'positive' results and full publication.

Moreover, more than half of the abstracts submitted to the CPDD did not report statistical results.

Conference proceedings do represent a valuable source of information to be included in systematic reviews, therefore there is growing concern about the reliability and quality of information published in these reports. A number of studies have highlighted the need for improvements in the reporting of conference abstracts and the abstracts of journal articles presenting the results of RCTs. In collaboration with the CONSORT (Consolidated Standards of Reporting Trials) Group, a checklist of essential items has been developed that authors should consult when reporting the main results of an RCT in any journal or conference abstract [9,10].

Some limitations of our study should be taken into account; we included only abstracts presented at one international conference because it is considered the most important international meeting in this field, and because there are no abstracts electronically and systematically available from other conferences. This may lead to an overestimation of the likelihood of publication, particularly for smaller studies or studies with null or negative results that may be present in less prestigious or local conferences. There is in fact a high probability that even fewer abstracts from these meetings end up published in full. It is also expected that the time lapse until publication is even longer for these abstracts. We may have touched only the tip of the iceberg of published or unpublished abstracts.

Moreover, we only searched two electronic databases (MEDLINE and EMBASE), and did not contact the authors of the studies for which we were unable to find the full publication. However, the two main electronic databases we searched provide 92% of the studies included in Cochrane reviews published in the area of drug and alcohol addiction [11]. In addition, our study does not account for other features of the reports that may influence time to publication, such as sample size and methodological quality.

In conclusion, our study confirms that abstracts that report null or negative results presented at conferences in the field of addiction are significantly less likely to be published than abstracts reporting positive results. If we consider that an additional bias could occur in the acceptance phase of abstract submission to conferences, possible publication bias should always be considered when conducting systematic reviews of the effectiveness of interventions for drug dependence in order to avoid biased results.

Making the registries of ongoing trials accessible may be one way to reduce publication bias. Researchers initiating randomized controlled trials should register trials to ensure the availability of trial results, independently of their full publication. Recently, the World Health Organization (WHO) launched an International Clinical Trials Registry Platform (ICTRP) with the mission of ensuring that a complete view of research is accessible to all those involved in health care decision making.

Eventually, given that conference proceedings are relevant for providing updated knowledge to be incorporated into systematic reviews of the effectiveness of health care interventions, more efforts should be put into ensuring that they fairly represent all research, regardless of the direction of their results.

## Abbreviations

CPDD: College on Problems of Drug Dependence; RCT: Randomised Controlled Trial; CCT: Controlled Clinical Trial; CI: Confidence Interval; SD: Standard Deviation; HR: Hazard Ratio; OR: Odds Ratio.

## Competing interests

The authors declare that they have no competing interests.

## Authors' contributions

MD e SV conceptualized the design of the study; SV extracted the data, wrote and updated the manuscript. VB analysed the data, LA, MD and CAP provided critiques and suggestions. All authors read and approved the final manuscript.

## Pre-publication history

The pre-publication history for this paper can be accessed here:

http://www.biomedcentral.com/1471-2288/9/23/prepub
